# A Flexible Capacitive Pressure Sensor Based on Ionic Liquid

**DOI:** 10.3390/s18072395

**Published:** 2018-07-23

**Authors:** Xiaofeng Yang, Yishou Wang, Xinlin Qing

**Affiliations:** School of Aerospace Engineering, Xiamen University, Xiamen 361005, China; yangxiaofeng@stu.xmu.edu.cn (X.Y.); wangys@xmu.edu.cn (Y.W.)

**Keywords:** sensor, aerodynamic pressure, ionic liquid, electrical double layer

## Abstract

A flexible microfluidic super-capacitive pressure sensor is developed to measure the surface pressure of a complex structure. The innovative sensor contains a filter paper filled with ionic liquid, and coated with two indium tin oxide polyethylene terephthalate (ITO-PET) films on the top and bottom, respectively. When external pressure is applied on the top ITO-PET film of the sensor mounted on the surface of an aircraft, the capacitance between the two ITO-PET films will change because of the deformation of the top ITO-PET film. The external pressure will be determined based on the change of the capacitance. Compared to the traditional pressure sensor, the developed sensor provides a high sensitivity of up to 178.5 nF/KPa and rapid dynamic responses for pressure measurement. Meanwhile, experiments are also conducted to study the influence of the thickness of the sensing film, sensing area, temperature, and humidity.

## 1. Introduction

Because of the characteristics of flexible flight and precise strike, hypersonic aircraft have always been an important research direction of space technology, since the mid-twentieth century. However, when the aircraft flies at a high speed in the atmosphere, the flow field will produce an aerodynamic effect. The shock wave will cause the boundary layer to be separated, resulting in the disturbance of an inviscid flow in the common boundary layer, which will influence the density, temperature, and composition of the airflow, and even produce the phenomenon of ionized gas molecules [1,2]. Therefore, it is of great significance to measure the aerodynamic pressure distribution in order to better understand the basic characteristics of the flow itself and its influence on the aircraft. According to the pressure distribution, the aerodynamic shape can be optimized.

In general, there are three main methods for measuring the distribution of aerodynamic pressure, the discrete pressure hole method [3,4,5,6,7,8,9], the pressure sensitive coating technology (PSP) [10,11], and the computational fluid dynamics (CFD) [12,13,14,15]. There are still many problems with these methods, both in the experimental measurement and theoretical analysis. The discrete pressure hole method is complex and its computation intensive. Meanwhile, the measurement hole will also affect the aerodynamic shape of the aircraft [4]. PSP technology is a relatively new technique for measuring the aerodynamic pressure on the surface. However, it is too complex and can only be used in wind tunnel experiments [10]. There are also some shortcomings in the CFD method. The numerical calculation method can lead to deformation, resulting in pseudo physical conclusions being produced [14,15]. Therefore, there is an urgent need to develop a new method to measure the distribution of aerodynamic pressure on the surface of an aircraft, for further exploring the aerodynamic shape of the aircraft [14].

Recently, flexible electronic sensors have been developed rapidly, including three kinds, piezoelectricity, piezoresistance, and capacitance [16,17,18,19,20,21,22,23,24]. Among the various sensors developed, capacitive sensors have a high stability, high electrical sensitivity, low power consumption, and fast response time, while piezoelectric and resistive sensors are widely applied because of their simple device and implementation [25,26,27]. An ionic skin through an ionic hydrogel, developed by Suo [28,29], can measure the changes in strains from 1% to 500%, and pressure as low as 1 kPa, with a small drift over many cycles. In addition, an ionic liquid sensor using ionic hydrogel as a sensing medium, in order to achieve an ultra-high sensitivity and flexibility pressure measurement, was developed by Pan [30,31,32]. However, the preparation process of the hydrogels is very complex, expensive, and the phase transition of the hydrogel at a high temperature will cause sensor failure.

A flexible microfluidic super-capacitive pressure sensor has been developed to measure the surface pressure in this paper. The sensor consists of a filter paper filled with ionic liquids, and coated with two indium tin oxide polyethylene terephthalate (ITO-PET) films on the top and bottom. Experiments are also conducted to study the influence of the thickness of the sensing film, sensing area, temperature, and humidity.

## 2. Sensing Principle

**Figure 1:**
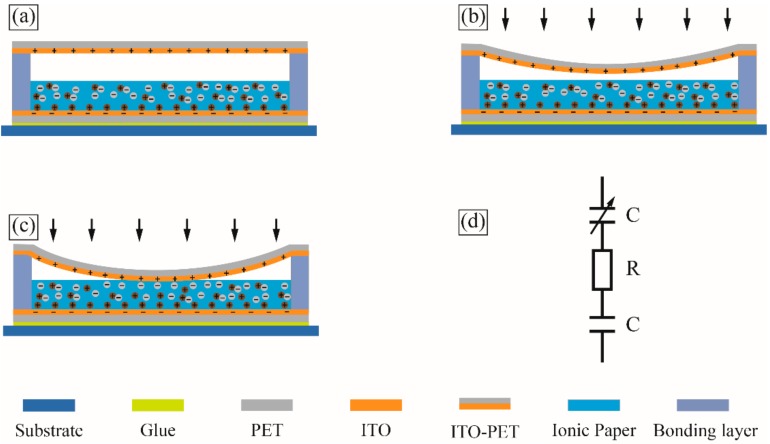
Sensing principle of the flexible ionic liquid super-capacitive pressure sensor. (**a**) No external pressure applied on the sensor. (**b**) Low external pressure applied on the sensor. (**c**) High external pressure applied on the sensor. (**d**) Equivalent circuit model of the sensor.

The schematic of the proposed flexible microfluidic super-capacitive pressure sensor is shown in Figure 1. The sensor contains a filter paper filled with ionic liquid, and is coated with two indium tin oxide polyethylene terephthalate (ITO-PET) films on the top and bottom, respectively. The sensing principle of the proposed sensor is based on the change of the capacitance between the two ITO-PET films. In a real application, the sensor is mounted on a surface of the host structure. The change of capacitance resulting from the deformation of the top ITO-PET film under external pressure is analyzed below.

When the external pressure is low, the top ITO-PET film does not contact the ionic liquid filter paper, as shown in Figure 1b. According to the classical thin-film bending theory, the change in capacitance of a circular sensing area can be described as follows [33,34]:

(1) $$C=\varepsilon_{0}\varepsilon_{r}\frac{A}{d}+\frac{3\varepsilon_{0}^{}\varepsilon_{r}AR^{4}(1-v^{2})}{16Eh^{3}d^{2}}P$$

where C is the capacitance of the sensor; *P* is the pressure applied on the top ITO-PET film; *R* is the radius of the sensing chamber; *ε*_0_ and *ε_r_* are the permittivity of vacuum and ionic liquid filter paper; *d* is the distance between the top ITO-PET film and the ionic liquid filter paper; A is the area of the sensing chamber; and *E*, *h* and ν are Young’s modulus, the thickness of the ITO-PET film, and Poisson’s ratio, respectively.

When the external pressure reaches a certain level, called the initial threshold, the top ITO-PET film makes ionic–electronic contact with the ionic liquid filter paper, as shown in Figure 1c. Notably, the ionic liquid filter paper, consisting of a substantial number of mobile cations, anions, and ionic liquid molecules, can form a unique super-capacitive layer in nano-scale, with exceptionally high unit-area capacitance, once in contact with the top ITO-PET film. This layer is known as the electrical double layer (EDL) [31]. As the external pressure goes up, the ionic liquid of the contact area has been compressed, and the contact area between the ionic liquid filter paper and the top ITO-PET film continues to increase, which leads to a further elevation of the EDL capacitance. According to the classical thin-film bending theory, the capacitance of a circular sensing area can be described as Equation (2), assuming an area radius, r, located in the center of the top ITO-PET film hang in the air.

(2) $$C=\frac{18R^{{}^{2}}P^{2}{\left(1-\nu^{2}\right)}^{2}-\sqrt{16Eh^{3}d}}{9C_{0}\pi R^{4}P^{2}{\left(1-\nu^{2}\right)}^{2}-8C_{0}\pi R^{2}\sqrt{16Eh^{3}d}}\;$$

where $C_{0}$ is the unit capacitance of the interfacial capacitance, and the meanings of the other symbols are the same as those in Equation (1).

According to the Gouy–Chapman–Stern and Kornyshev models, the EDL can be treated as a capacitive sensor when no electrochemical reaction occurs at the interface of the ITO-PET film and ionic liquid filter paper. As shown in Figure 1d, the entire sensor can be regarded as a variable capacitor in series, with a resistive element from the ionic liquid filter paper and a fixed capacitor from the interface of the bottom ITO-PET film and ionic liquid filter paper [30,35,36].

## 3. Experimental Investigation

### 3.1. Materials and Apparatus

Advantec filter papers (No. 1, Roshi Kaisha., Ltd., Tokyo, Japan) were employed in experiment. These filter papers have a thickness of 0.15 mm and a basic density of 90 g/m^2^. Ionic liquid 1-butyl-3-methylimidazolium bis-(trifluoromethyl)-imide (Shanghai Chengjie Chemical Co. Ltd., Shanghai, China) was selected as the sensing material. Polyethylene terephthalate (PET) coated with a 100 nm thick layer of indium tin oxide (ITO, Mianyang Prochema Commercial Co., Mianyang, China) was used for the electrode layer. The bonding layer was made of double-sided adhesive type (VHB 4905, 3M). A WK6500B (Shenzhen Wenke Electronics Co., Ltd., Shenzhen, China) impedance analyzer was utilized for measuring the capacitance. A KD-II 10/100 N (Shenzhen Kaiqiangli Technology Co., Ltd., Shenzhen, China) mechanical testing apparatus was used for precisely applying the pressure load on the sensor. A constant temperature and humidity incubator (Shanghai Baixin Instrument and Equipment Factory, Shanghai, China) were used to control the testing temperature and humidity.

### 3.2. Sensor Design

**Figure 2:**
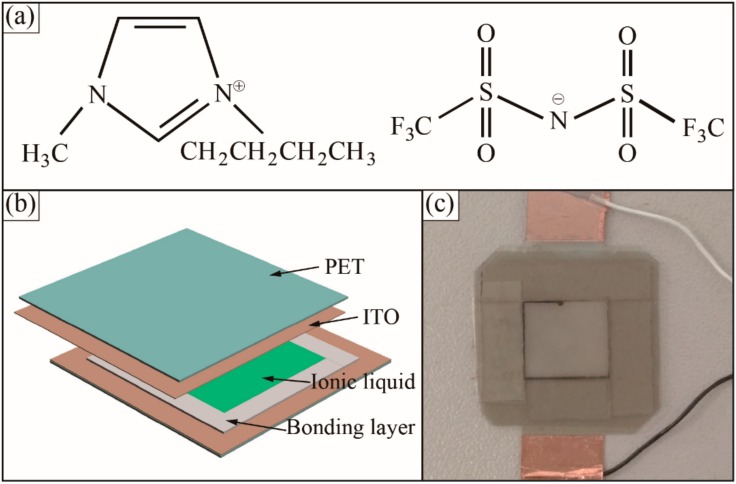
Flexible ionic liquid super-capacitive pressure sensor. (**a**) Chemical structure of ionic liquid. (**b**) Schematic diagram of the flexible sensor. (**c**) Actual fabricated flexible ionic super-capacitive pressure sensor.

In order to achieve a reversible ionic liquid-ITO electrode contact, a surface modification technique has been adapted to enhance the ITO electrode surface hydrophobicity. A fluorinated separation layer of trimethoxysilane, with a proportion of 3% in IPA was spun at 3000 rpm for 40 s to coat the ITO electrode, and dried at atmospheric pressure in an oven at 80 °C for 1 h. Then, the double-sided adhesive tape was attached on the ITO substrate to form the designed sensing chamber. The filter paper was immersed in ionic liquid for 2 min and was then put on a napkin to absorb the excess ionic liquid. The chemical structure of the ionic liquid was shown in Figure 2a. After transferring the ionic liquid paper into the sensing chamber, the sensing chamber was then sealed with another ITO-PET film. For ease of use, conductive epoxy was used for making the wire connection. Figure 2b,c shows a schematic diagram and prototype of the flexible sensing device in a package of 15 mm × 15 mm × 5 mm.

## 4. Results and Discussion

### 4.1. Response of Capacitance with Driving Frequency

**Figure 3:**
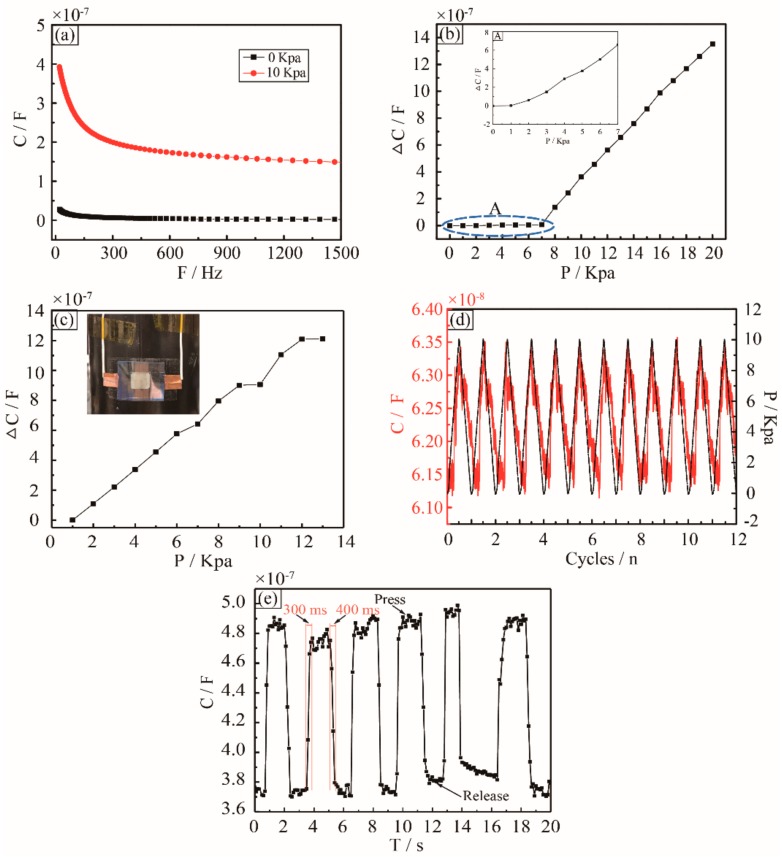
Characterization of the pressure sensing performance of the flexible ionic liquid super-capacitive pressure sensor. (**a**) The capacitance changes with driving frequency sweep from 20 Hz to 1.5 KHz at 0 KPa and 10 KPa, respectively. (**b**) Relative rate of change in capacitance of the sensor as a function of different pressure. The inset shows that the relative rate of change in capacitance of low pressure region A. (**c**) Relationship of the change in capacitance of the sensor attached on a circular tube and pressure applied. The inset shows the actual sensor attached on the circular tube. (**d**) The relationship of pressure and capacitance of the sensor cyclically compressed by 10 KPa. (**e**) The capacitance variation of the sensor under a square shaped pressure applied.

The developed sensor used for testing was fixed on an acrylic plate. In order to perform the experiment in the lab, a squared rigid flat plate with the area of 25 mm^2^ attached on the top ITO-PET membrane was used to convert a point force into a distributed force to simulate the distributed air pressure applied on the sensor. To characterize the sensor sensitivity, this point force was applied to the center of the flat plate. The pressure was calculated by dividing the force by the area of the membrane. The change of capacitance was recorded by the impedance analyzer when the pressure was applied on the sensor. Because the permittivity decreases with the decrease of the driving frequency, the response of the sensor varied with the frequency. Driving frequencies from 20 Hz to 1.5 KHz at 0.5 alternating current (AC) voltage were selected for the testing. Figure 3a shows the capacitance of the sensor as a function of the driving frequency at the pressure of 0 Pa and 10 KPa. It can be seen that the capacitance variation of the sensor decreases when the driving frequency increases, and it has maximum value at 20 Hz. Therefore, within the same capacitance variation range, different pressure sensing ranges can be realized by changing the driving frequency. Considering energy consumption, actual measurement situation, and, also, to avoid electrochemical reactions, a 20 Hz driving frequency at 0.5 AC voltage was chosen for the testing mentioned hereafter.

### 4.2. Relation between Pressure and Change of Capacitance

Figure 3b illustrates the change of capacitance of the sensor with different pressure applied on the sensing membrane, giving the thickness of the sensing membrane at 50 μm and the sensing area at 10 mm × 10 mm. As the pressure was applied on the sensor, the capacitance of the sensor was measured by the impedance analyzer in real-time. Figure 3b shows the relationship between the change of capacitance and the actual pressure applied on the sensor. The slope rate of the curve was defined as the sensing sensitivity. It is clear that the change of capacitance depends on the pressure. Firstly, when the pressure is below 7 KPa, the top ITO-PET film does not make contact with the ionic liquid filter paper, and the capacitance increases according to the distance narrowing between the top ITO-PET film and the ionic liquid filter paper. The sensing sensitivity is 1.5 nF/KPa, as shown in Figure 3b. When the pressure is in the range of 7 KPa to 20 KPa, the sensing sensitivity reaches 108.2 nF/KPa. The reverse ion in the ionic liquid forms a double capacitance layer on the surface of the electrode, resulting in a sharp increase in the capacitance. When the pressure is continuously increased, the sensitivity decreases because of the reduced mechanical deformation of the ITO-PET film, where the small deformation limit has been exceeded. However, if the actual surface pressure of the aircraft may be over 50 KPa, the current sensor cannot meet the actual operating condition of the aircraft, and we should do some further research to improve the sensing range of the current sensor in the future work.

The flexible of the sensor allows it to conform readily to a curved surface. We attached the flexible capacitive sensor on a circular tube with a 33 mm in radius to investigate the influence of bending to the pressure measurement of the sensor. As shown in Figure 3c, when the external pressure is applied on the sensor, the capacitance of the sensor increases rapidly. The sensing sensitivity is 110.2 nF/KPa, but the sensing range decreases to 11 kPa. That is because when the sensor was attached on the curved surface, the ionic liquid filter paper was bended and contacted the top ITO-PET electrode, which can be seen in Figure 3c.

A quasi-static cycling load with a maximum pressure of 10 KPa at frequency 0.05 Hz was applied on the sensor. The capacitance of the sensor and the pressure that was applied were recorded, as shown in Figure 3d. Both the capacitance and pressure curves are stable, and they match each other well. As described above, the pressure of 10 KPa is enough to make the ITO-PET film contact the ionic liquid filter paper. It is observed that once the pressure is applied on the top of the ITO-PET film, the film deforms and makes contacts with the ionic liquid filter paper to make the capacitance of the sensor increase immediately, and the capacitance returns back to the original value after unloading the pressure.

As shown in Figure 3e, a square shaped pressure was applied on the sensor, which is attached on the circular tube, and the capacitance is stable and consistent with the square pressure applied on the sensor. The response and recovery times of 0.3 s and 0.4 s can be extracted from the upstroke response of the sensor readout signal. Overall, the sensor is able to respond well to the static and dynamic pressure applied on it.

### 4.3. Effect of the Thickness of the Sensing Membrane

**Figure 4:**
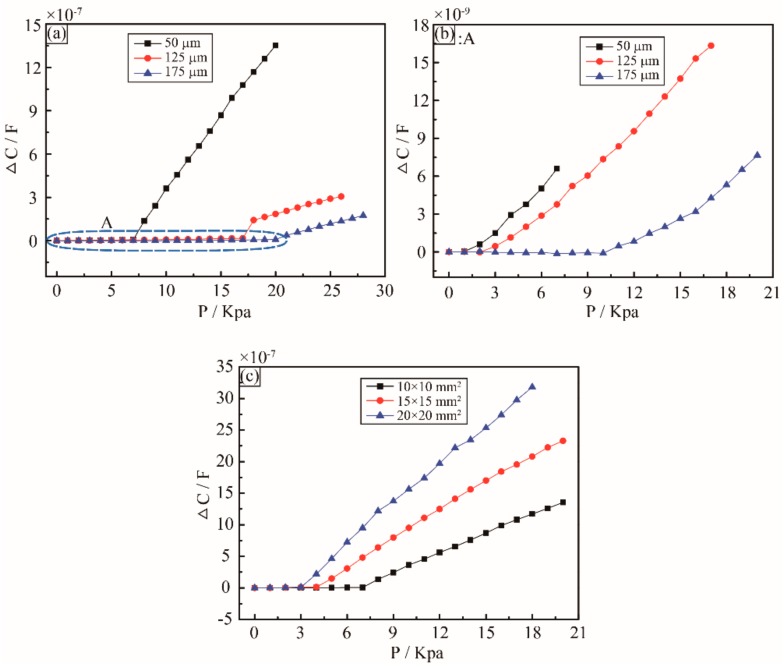
Influence of geometrical parameters on the sensor sensitivity. (**a**) Different thickness of the sensing membrane, 50 μm, 125 μm, and 175 μm. (**b**) The enlargement of the low pressure region A in Figure 4a. (**c**) Different areas of the sensing chamber, 10 mm × 10 mm, 15 mm × 15 mm, and 20 mm × 20 mm.

From the experimental result, it is clear that the sensitivity of the sensor is inversely proportional to the thickness of the sensing membrane. Figure 4a shows the relationship between the change of capacitance and the actual pressure on the sensor, and the thickness of the sensing membrane varying from 50 μm to 175 μm. With the thickness of the sensing membrane increasing from 50 μm to 175 μm, the sensitivity decreases from 1.5 nF/KPa to 1 nF/KPa at the low pressure below 7 KPa, as shown in Figure 4b: A, and significantly decreases from 108.2 nF/KPa to 18.5 nF/KPa at the pressure range of 7 KPa to 28 KPa. The detective pressure range is also influenced by the thickness of the sensing membrane. When the thickness of the sensing membrane varies from 50 μm to 175 μm, the maximum pressure being detected increases from 20 KPa to 28 KPa. According to the different sensing applications, different thickness of the sensing membrane shall be used.

### 4.4. Effect of Area of the Sensing Chamber

Notably, the geometrical parameters of the sensing chamber also influence the sensor sensitivity and detective range. As shown in Figure 4c, three different areas of the sensing chambers have been characterized and compared. It is clear that the sensor sensitivity is directionally proportional to the area of the sensing chamber. The area of the sensing chamber increases from 10 mm × 10 mm to 20 mm × 20 mm, and the corresponding sensor sensitivity raises from 108.2 nF/KPa to 178.5 nF/KPa, but the detective range decreases from 20 KPa to 18 KPa. For the case of the 20 mm × 20 mm sensing area, the sensitivity is much higher than most of the existing capacitive sensors, as summarized in Table 1 [19,37,38,39,40].

### 4.5. Effect of Temperature and Humidity

**Figure 5:**
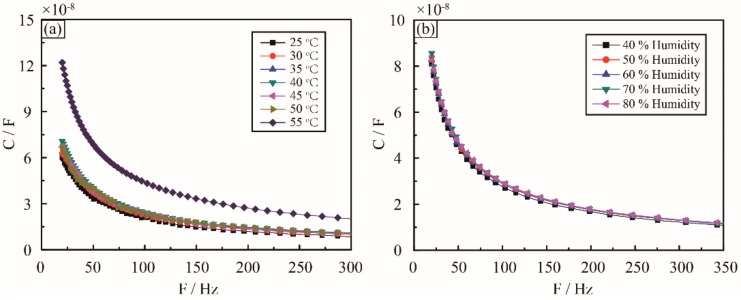
Effect of the environment factors. (**a**) Capacitance change with frequency at temperature range from 25 °C to 55 °C. (**b**) Capacitance follows the frequency at humidity range from 40% to 80%.

Importantly, the actual service environment of the sensor is very complex, for example, environmental factors, such as humidity and temperature, are often varying in time. Because of the high boiling point, low vapor pressure, and non-flammability of the ionic liquid, the environmental temperature fluctuation only a poses minor influence on the interfacial capacitance. As shown in Figure 5a, the capacitances of the sensor were measured in the temperature range of 25 °C to 55 °C. It is clear that the capacitance is stable, with less than 2% variation from 25 °C to 50 °C. This is likely due to the stabilization of the physical and chemical properties as well as the ionization of the ionic liquid in the temperature range. When continuously increasing the temperature, the EDL capacitance rises dramatically because of the increase in the ionization of the ionic liquid with the temperature above 50 °C. It should be noted that the actual service environment of the aircraft is very complex, the surface temperature may be over 100 °C or even higher, and we should do further research to improve the temperature adaptability of the current sensor in the future work.

Furthermore, the performance of the sensor at different humidity levels has been investigated. As shown in Figure 5b, the capacitance is very stable at different humidity levels ranging from 20% to 80%. This is because the ionic liquid does not react with water at low-voltage AC signal, and the ionization of the ionic liquid is not sensitive to the humidity.

## 5. Conclusions

A flexible super-capacitive sensor with a simple architecture and high sensitivity was developed. The experimental results show that the sensor responds well at both static and dynamic pressure, with a high sensitivity, good linearly, and repeatability. Compared to a traditional pressure sensor, this sensor provides a high sensitivity of up to 178.5 nF/KPa, and a good dynamic response for pressure measurement. Moreover, the temperature and humidity effect on the performance of the senor were also investigated. The capacitance of the sensor with selected materials is stable from 25 °C to 50 °C, and not sensitive to humidity.

Overall, the developed innovative sensor has significant potential for low cost and reliable surface pressure measurement, but there are still some issues that need to be further improved for the practical application. For example, the performance of the sensors should be further tested and verified in a real air pressure environment, the size of sensors should be made as small as possible to reduced influence of the bending, and the sensing range and temperature resistance of sensors should be further researched.

## Figures and Tables

**Table 1:** Comparison of the sensitivity of capacitive pressure sensors.

Types	Dielectric Layer	Sensitivity	Sensing Range
Silicon-based	Air [37]	3.47 fF/KPa	0–30 Kpa
Elastic material-based	PDMS [38]	7.2 pF/KPa	0.5–2.5 KPa
	CNT/PDMS [39]	0.55 nF/KPa	0–0.03 KPa
	Pyramid PDMS [19]	18.6 fF/KPa	0–2 KPa
	Porous PDMS [40]	0.1 pF/KPa	0–0.02 KPa
Microfluidic-based	Hydrogel [29]	0.1 pF/kPa	0–40 KPa
	Nanofiber [30]	114 nF/Kpa	0–1 KPa
	Air/ionic liquid filter paper [this work]	178.5 nF/kPa	7–18 KPa
